# The development of an antimicrobial EVD catheter to protect against multi-resistant hospital “superbugs”

**DOI:** 10.1186/1743-8454-7-S1-S46

**Published:** 2010-12-15

**Authors:** Waheed Ashraf, Roger Bayston, Oxana Stevenson

**Affiliations:** 1 BRIG, Division of Orthopaedic and Accident Surgery, Nottingham University Hospitals QMC, Nottingham, NG7 2UH, UK

## Background

External Ventricular Drainage (EVD) infections are a serious complication, and the period of risk extends until the device is removed. The causative bacteria of EVD and shunt infections are similar but there are more multi – resistant strains such as MRSA and gram negative bacteria in EVD infections, particularly when the patient is in intensive care. To counter this, we have development an antimicrobial EVD catheter with a broad spectrum to protect against these multi-resistant bacteria. Thus, the aim of this study was to evaluate the antimicrobial activity of this catheter against multi-resistant Staphylococcus epidermidis, MRSA, and gram negatives using clinically predictive in vitro tests.

## Materials and methods

Medical grade silicone catheter tubing (Codman) was impregnated with antimicrobials: 1% triclosan, 1% trimethoprim and 0.2% rifampicin respectively. Three methods were used to evaluate the antimicrobial activity of the catheter. The Serial Plate Transfer Test (SPTT) is a screening test for duration of antimicrobial activity and to monitor resistance. Impregnated catheter segments were placed onto agar plates seeded with bacteria and incubated. Segments were removed daily and placed on fresh plates and reincubated. The inhibition zone was measured across the short axis. This was repeated until no inhibition zones were seen. The time taken to kill 100% of bacteria attached to catheter segments (tK100), was determined by first coating the catheter segments with a protein conditioning film, then allowing the bacteria to adhere to plain and antimicrobial catheter segments and incubating them. Samples were retrieved daily, sonicated to remove the adherent bacteria, and the sonicate cultured quantitatively to detect bacterial growth. Thirdly, a simulated in vitro model was used to determine the ability of the antimicrobial catheter to resist successive bacterial challenges every 14 days under constant perfusion, designed to mimic the CSF flow.

## Results

The SPTT showed duration of antimicrobial activity for more than 80 days. The tK100 showed that it takes between 24-48 hours to kill all the bacteria attached to the catheter. The in vitro model showed that the catheter protected against bacterial colonization after 7 successive challenges (ie more than 80 days), without developing resistance.

## Conclusions

The catheter demonstrated a broad spectrum of antimicrobial activity and a prolonged duration of activity against multi- resistant bacteria. The clinically predictive tests indicate that the catheter is likely to reduce significantly EVD infections caused by multi-resistant superbugs found among patients on intensive care and high-dependency units.

## Competing interests

RB holds a patent for the catheter described, and receives consultancy fees from Codman but not for personal gain. WA and OS have no interests to declare.

